# Protocol of a multicentric prospective cohort study for the VALIDation of the IBD-disk instrument for assessing disability in inflammatory bowel diseases: the VALIDate study

**DOI:** 10.1186/s12876-020-01246-7

**Published:** 2020-04-16

**Authors:** C. Le Berre, A. Bourreille, M. Flamant, G. Bouguen, L. Siproudhis, M. Dewitte, N. Dib, E. Cesbron-Metivier, T. Goronflot, M. Hanf, P.-A. Gourraud, E. Kerdreux, A. Poinas, C. Trang-Poisson

**Affiliations:** 1 Institut des Maladies de l’Appareil Digestif, Nantes University Hospital, Nantes, France; 2 Service des Maladies de l’Appareil Digestif, Rennes University Hospital, Rennes, France; 3 Service de Gastroentérologie, Angers University Hospital, Angers, France; 4 Clinique des Données, Nantes University Hospital, Nantes, France; 5 Centre d’Investigation Clinique, Nantes University Hospital, Nantes, France; 6 Direction de la Recherche Clinique, Nantes University Hospital, Nantes, France

**Keywords:** Inflammatory bowel diseases, Disability, Quality of life, IBD disk, IBD-disability index, Patient-reported outcomes

## Abstract

**Background:**

Inflammatory Bowel Diseases (IBD) affect psychological, family, social and professional dimensions of patients’ life, leading to disability which is essential to quantify as part of Patient-Reported Outcomes (PROs) newly included in the targets to reach in IBD patients. Up to now, the IBD-Disability Index (IBD-DI) was the only validated tool to assess disability, but it is not appropriate for use in clinical practice. The IBD Disk was developed, a shortened and self-administered tool, adapted from the IBD-DI, in order to give immediate representation of patient-reported disability. However, the IBD Disk has not been validated yet in clinical practice. The aims of the VALIDate study are to validate this tool in a large population of IBD patients and to compare it to the already validated IBD-DI.

**Methods:**

The VALIDate study is an ongoing multicentric prospective cohort study launched in April 2018 in 3 French University Hospitals (Nantes, Rennes, Angers), with an objective to reach a sample of 400 patients over a period inclusion of 6 months. Each patient will fill in the two questionnaires IBD Disk and IBD-DI at baseline, then between 3 and 12 months later, during a follow-up visit. Clinical and socio-demographic data will also be collected. During these two consultations, gastroenterologists and patients will evaluate disease activity thanks to a semi-quantitative 4-grade scale, named respectively PGA (Physician Global Assessment) and PtGA (Patient Global Assessment). This cohort will allow to evaluate the validity of the IBD Disk with respect to the IBD-DI in order to generalize its use for clinical practice. Other psychometric criteria of the IBD Disk will also be analysed as its reliability or its discriminant capacity. Close attention will nonetheless be needed to minimize the number of lost to follow-up patients between baseline and follow-up.

**Discussion:**

The VALIDate study is the study designed to validate the IBD Disk, a visual tool easily useable in daily practice to assess disability in IBD patients. The results of this trial should enable the diffusion of this tool.

**Trial registration:**

The trial is registered in ClinicalTrials.Gov with registration number NCT03590639. First posted: July 18, 2018.

## Background

Inflammatory Bowel Diseases (IBD), comprising Crohn’s Disease (CD) and Ulcerative Colitis (UC), are chronic gastrointestinal disorders leading to a progressive and cumulative digestive tract damage [1–3]. They often are responsible for many digestive and extra-intestinal symptoms (diarrhoea, abdominal pain, rectal bleeding, joint pain) but can also affect psychological, family, social and professional dimensions of patients’ life, leading to decrements of function or disability [4–13], without taking into account the high societal costs directly or indirectly linked to this disease-related burden [14].

Over the past decade, due to an increasing attention to the patient’s voice in all aspects of health care, the concept of “Patient-Reported Outcomes” (PROs) has been developed, defined as any report that comes directly from a patient about a health condition or its treatment, without interpretation of the patient’s response by a clinician or anyone else [15]. PROs have become an integral part of the endpoints evaluated in IBD clinical trials [16–18], strongly recommended by the United States Food and Drug Administration (FDA) [19], as PROs are now considered as real therapeutic targets [20, 21]. Indeed PRO bring benefit for both patients and health care professionals improving physician-patient communication and patient’s quality of life. PROs foster patient-centered care; patients feel better able to communicate their experience to the team, it improves communication and shared decision-making, and facilitates multidisciplinary team care [22].

Several disease-specific and generic tools have been developed to assess PROs in IBD patients: some are devoted to quality of life like the Inflammatory Bowel Disease Questionnaire (IBDQ) and its short version (SIBDQ) [23–25], while others permit to assess fatigue (Functional Assessment Chronic Illness Therapy–Fatigue FACIT-F) [26], work productivity [27–29], as well as depression and anxiety [30–34]. More recently, an IBD Distress Scale has been constructed to evaluate IBD-specific distress [35].

A French cohort of 1185 IBD patients has shown that half of the patients reported poor quality of life with a SIBDQ< 45 (53.3%), severe fatigue with a FACIT-F < 30 (47.4%) and/or depression (HAD-D > 7: 49.4%). Around one third of the patients reported anxiety (HAD-A > 7: 30.3%) and/or moderate (22.4%) or severe (11.9%) disability [36].

Health-related quality of life has been explored since the 1970s in IBD,[37] but this concept is subjective. Furthermore, none of the existing tools measuring quality of life in IBD was developed according to the FDA guidance [19]. Conversely, according to the World Health Organization (WHO), disability is an objective umbrella term for impairments, activity limitations and participation restrictions, which is essential to quantify because physicians frequently underestimate disease-related disability in IBD patients [38–40]. However, before 2015, when compared to quality of life, there was no specific tool dedicated to disability in the field of IBD, in contrast to other chronic diseases especially inflammatory diseases, like psoriasis [41, 42], rheumatoid arthritis [43, 44] or multiple sclerosis [45, 46].

Yet, several surveys have shown disparities between patients’ and gastroenterologists’ perceptions of the impact of IBD on patients’ lives [38–40, 47]: indeed, physicians often under-estimate disease burden while disabled patients prefer to accommodate their lives to their disease rather than act to optimize therapy [48]. Thus, there is a need for reliable tools to assess disability in IBD patients, in order to improve communication between patients and gastroenterologists, thereby enhancing treatment adherence [49–52].

In 2015, a new index has been developed, based on the World Health Organization’s International Classification of Functioning, Disability and Health (ICF) [53], and specifically devoted to the assessment of disability in IBD patients [54]. This index, called Inflammatory Bowel Diseases Disability Index (IBD-DI), comprises 14 questions and ranges from 0 to 100. The IBD-DI has been validated in a French population based-cohort for use in clinical trials and epidemiological studies, and showed high internal consistency, interobserver reliability and construct validity, and a moderate intra-observer reliability [55]. Despite its robustness, this index requires to be filled in attendance of a health care professional and seems to be difficult to apply in daily practice.

In this context, in 2017, Ghosh et al. have developed the IBD Disk, a shortened, self-administered and visual tool, adapted from the validated IBD-DI, in order to give immediate representation of patient-reported IBD-related disability [56]. The IBD Disk is largely inspired by the PsoDisk, a visual instrument developed in 2012 by the dermatologists to assess disability in patients suffering from psoriasis [57], which has been validated since in an Italian cohort of 320 patients [58]. The IBD Disk was developed using a consensus-based process to select items from the IBD-DI that are most likely to be important in assessing patient’s disease burden and are relevant to both patient and physician.

This new tool should allow the gastroenterologist to rapidly assess the patient’s disability at a given time, but could also be used to follow changes in disease burden over time, thereby monitoring treatment efficacy with a more global picture of the patient’s health.

However, the IBD Disk has not been validated yet in national clinical practice. In 3 hospitals located in the West region of France (Nantes, Angers and Rennes), we begin to use it, as a physician help. But it was crucial to know if this questionnaire was reproducible and if it could correlate with what physicians see and what the patient describes.

After a comprehensive description of the study sample which will allow to better describe French IBD-patients’ characteristics, the primary objective of the VALIDate study will be to validate the IBD Disk and to evaluate its consistency with the already validated IBD-Disability Index.

Other investigations will allow to assess the reliability of the tool, its variability over time, and its correlation with the clinico-biological activity of the disease, and with the assessment of the disease activity by the patient and the physician.

## Methods and design

### Study design and setting

The VALIDate study is a prospective multicentric cohort study led in three West-French University-affiliated Hospitals (Nantes, Rennes, Angers), Nantes being the principal investigator centre. The recruitment of patients has begun in April 2018 and will be carried out over a period of 6 months, and the patients’ follow-up will be led until August 2019.

There is no unanimously accepted formula to define a number of subjects in a questionnaire validation phase. It is nevertheless common to have samples between 200 and 500 individuals. The sample size was determined according to feasibility criteria [59] which suggest a total of 10 subjects per item in the questionnaire needed for validation. The IBD Disk questionnaire consists of 10 items, hence requiring a minimum sample size of 100 subjects. The high number of patients expected to be lost at follow-up made it advisable to increase the sample to 400 patients (approximately 130 patients per site).

**Fig. 1:**
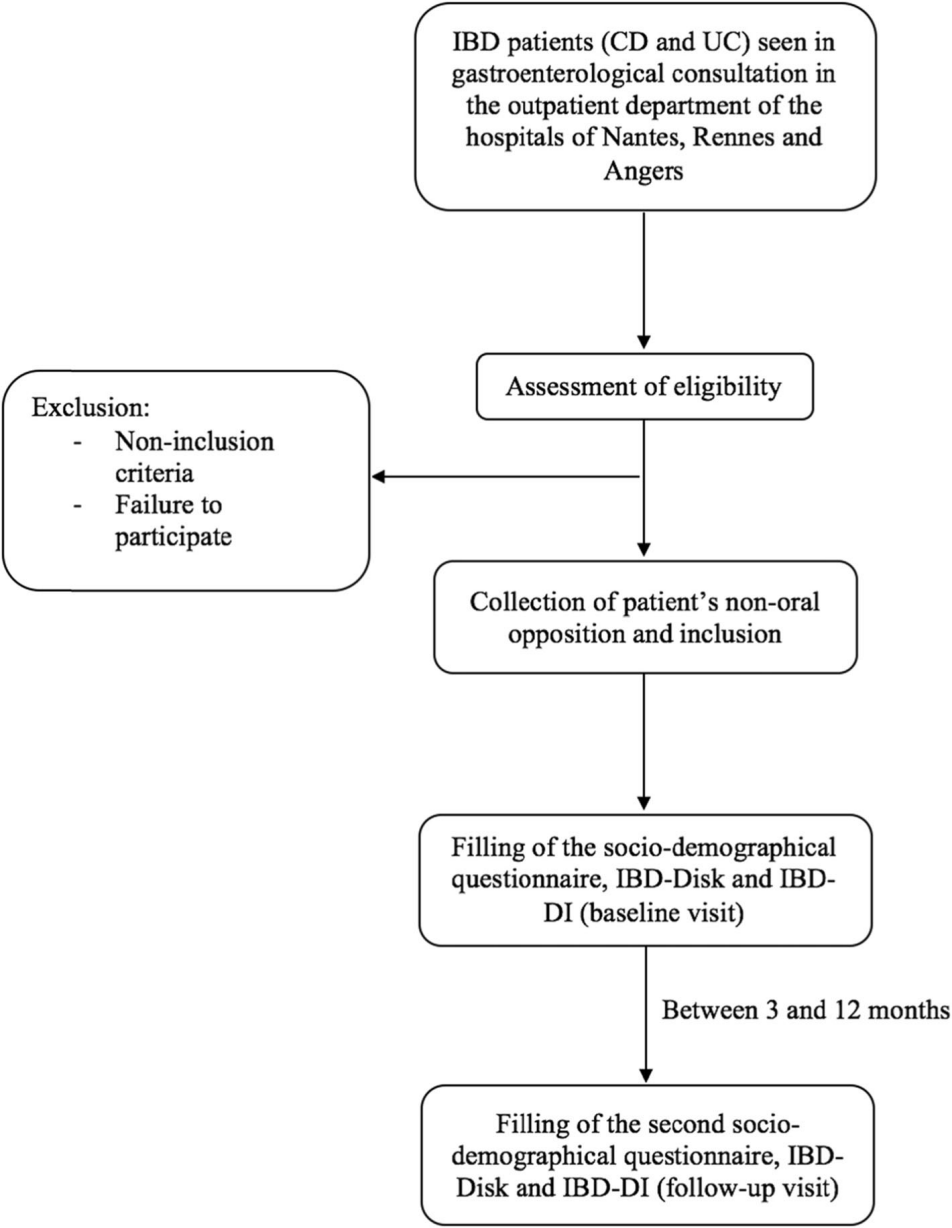
Flow chart of the VALIDate study. Patients will be recruited consecutively in the three outpatient departments of the hospitals of Nantes, Rennes and Angers. If they meet the inclusion criteria, patient information will be given orally and in writing, then the physician will collect patient’s oral non-opposition. Both IBD-Disability Index and IBD Disk questionnaires will be filled by all included patients at baseline visit, then between 3 and 12 months after baseline, during a routine follow-up visit. Patients will also respond to a socio-demographical questionnaire during both visits

Patients will be recruited consecutively in the three outpatient departments of the hospitals of Nantes, Rennes and Angers. Figure 1 displays the flowchart representing patients’ course during the study.

### Study population

Patients of both sexes, aged over 18 with an established diagnosis of IBD (Crohn’s Disease, Ulcerative Colitis or IBD unclassified), may be included in the VALIDate study. No limits are set concerning prior, planned or concomitant therapies. Non-inclusion criteria include: ongoing pregnancy or breast-feeding woman, vulnerable people i.e. adults under a legal protection regime (guardianship, trusteeship, judicial safeguard), insufficient command of French language and relevant psychiatric comorbidities (both of them making the self-questionnaire difficult to fill in), and uncertain IBD diagnosis.

There will be no emergency inclusion. Patients can participate to another clinical trial at the same time.

At baseline, patient information will be given orally and in writing (Annex 1), then the physician will collect patient’s oral non-opposition. Indeed, as questionnaires are given in common practice in the 3 centres, this trial does not correspond to the French legislation of the Loi Jardé (Article L1121-4 amended by Ordinance No. 2016-800 of 16 June 2016 - Art. 1). The VALIDate study is considered as a non-interventional study based on data collection of consultations carried out during follow-up care of patients; no authorization is requested from the French regulatory authorities. Patients will be informed that the data collected in their medical records will be used in this study. Their oral non-opposition will be collected after reading the information letter (Annex 1) and noted in the medical record as required. This study was approved by the local Research Ethics Committee (GNEDS) on 18 May 2018.

The trial is registered in ClinicalTrials.Gov with registration number NCT 03590639, registered 18 July 2018.

### Test methods

Before the beginning of the study, a preliminary step before the administration of the IBD Disk was its translation in French, because the tool was created in English. As the IBD-DI had previously been translated in French [55] (Annex 2), as recommended by Beaton et al. [60], a medical board from AbbVie translated the IBD Disk avoiding the four first stages of cross-cultural adaptation, considering that the items included in the tool were directly derived from the items of the IBD-DI. The IBD Disk was then tested by two gastroenterologists and one IBD specialist nurse in 74 consecutive IBD patients between November 2017 and February 2018, demonstrating its good acceptability and clarity. Annex 3 shows the final version of the French IBD Disk used in this study.

The IBD Disk consists of 10 questions, exploring abdominal pain, regulating defecation, interpersonal interactions, education and work, sleep, energy, emotions, body image, sexual functions and joint pain. All included elements, except “sexual functions”, were part of the validated IBD-DI. “Sexual functions” was included from the comprehensive ICF score set [5]. Each answer is marked on a 11-point visual analog scale, from 0 to 10, 0 corresponding to “absolutely not” (no complaint) and 10 to “definitely yes” (maximal complaint). The points are then connected and a polygon is obtained, whose area may immediately be interpreted as the size of the disease-related burden.

Both IBD-DI and IBD Disk questionnaires will be filled by all included patients at baseline visit, then between 3 and 12 months after baseline, during a routine follow-up visit.

Furthermore, 70 patients included in the hospital of Nantes will be asked to complete again the IBD Disk 7 days after baseline visit and to send it to the principal investigator by post (provided stamped envelope), in order to evaluate the test-retest reliability of the score. A phone contact may be made to the patient at the end of the 7-day period if he has forgotten.

During these two consecutive consultations, both gastroenterologists and patients will also evaluate disease activity thanks to a semi-quantitative 4-grade scale, named respectively PGA (Physician Global Assessment) and PtGA (Patient Global Assessment), and broken down as: inactive, mild, moderate, or severe disease.

Table 1 corresponds to the timetable of the VALIDate study.

**Table 1:** Timetable of the study

Action	Baseline (inclusion visit)	7 days (for the sub-sample of 70 patients taking part in the test-retest)	14 days (for the patients who forgot to send the IBD Disk again)	3 to 12 months after baseline (follow-up visit)
Patient information and collection of oral non-opposition	X			
Collection of socio-demographic data	X			X
Collection of clinical data	X			X
Physical examination	X			X
IBD-DI filling	X			X
IBD Disk filling	X	X^a^		X
Physician Global Assessment	X			X
Patient Global Assessment	X			X
Biological measures: CRP Faecal calprotectin	X X			X X
Phone contact			X	

^a^Sent by post

### Variables

Clinical data will be collected by the gastroenterologist at baseline including: type of IBD (Crohn’s Disease, Ulcerative Colitis or IBD unclassified), date of diagnosis, disease location and behaviour according to Montreal classification [61], history of intestinal resection or perianal surgery, ongoing disease-related treatments, disease activity index (Harvey Bradshaw Index HBI for CD, Mayo clinical sub-score for UC), and biological markers if available (C-reactive protein CRP and faecal calprotectin). Similar data will be collected again at follow-up visit except for unchanged variables (type of IBD, date of diagnosis).

Socio-demographic data will also be collected including: age at diagnosis, age at baseline, sex, professional status, family status, number of children, smoking status, sporting activity, special diet (low residue, low gluten, low lactose), symptomatic medication use (antidiarrheal, intestinal adsorbent, antispasmodic), anxiolytic/anti-depressant use. At the time of follow-up visit, the patient will also specify if he has consulted his general practitioner or been hospitalized because of IBD complications since the baseline visit.

### Data management

The principal investigator is committed to maintain the confidentiality of patients involved in the study. In this respect, all data from the 3 participating centres will be processed by a single person (CLB) who will generate an anonymity code for each participant, in order to create an electronic dataset without any information on patients’ identity. That same person will keep a separate document that links the anonymity code to subjects’ identifying information; this file will be locked in a separate location and its access will be strictly restricted to the principal investigator. Transmission of a person’s data for research purposes will therefore only be possible subject to the application of this coding system; the presentation of the research results must exclude any direct or indirect identification.

### Analyzes

Firstly, a comprehensive descriptive analysis will allow to present the clinical and sociodemographic characteristics of IBD patients in our study. Continuous data will be presented as means (± standard deviations SD) or medians (interquartile ranges IQR), depending on their distribution. Categorical data will be presented using numbers (%). These results will be presented either as a full article or in addition to another analysis.

Thanks to this cohort, several intrinsic characteristics of the IBD-Disk will be able to be evaluated, such as validity, reliability, reproducibility or its discriminating power. The results of these analyzes will make it possible to give an opinion as to the generalization of the IBD-Disk tool in clinical practice to evaluate the level of incapacitation that brings the disease to the patient on a daily basis.

Validity expresses the degree to which a measurement measures what it purports to measure [62]; several varieties will be studied (“floor/ceiling effects”, discriminant validity, and concurrent validity thanks to correlations between IBD Disk and IBD-DI questionnaires). Reliability refers to the degree to which the results obtained by a measurement can be replicated. IBD Disk’s reliability will be studied using Cronbach’s coefficient alpha, a statistic calculated from the pairwise correlations between items. To assess IBD Disk’s reproducibility, we will rely on the sub-sample of 70 patients who will have completed again the IBD Disk 7 days after the first visit. It will allow us to evaluate the capacity of the IBD Disk to remain stable if it is administered twice to the same person in a short time interval. Based on Walter et al. [63], with an expected reliability of the intraclass correlation coefficient (ICC) between the test/re-test of IBD Disk of 0.80, an alpha risk of 5% and a subset of 70 patients, our study design will give us a power of 91% to show a minimum acceptable reliability of the ICC of 0.60.

## Discussion

The VALIDate study is a prospective multicentric cohort study with the aim of validating the IBD Disk in a large population of IBD patients and to compare it to the already validated IBD-Disability Index (IBD-DI).

This reflects the growing consideration for patients’ well-being for about 10 years in the IBD field. Besides, Patient-Reported Outcomes (PROs) and among them disability, have been included in the STRIDE consensus [20] initiated by the International Organization for the Study of Inflammatory Bowel Diseases (IOIBD) in order to determine therapeutic goals in IBD in the context of “treat-to-target” strategies that could be used in clinical practice [64]. However, up to now, the IBD-DI was the only tool validated for the assessment of disability in IBD patients, however it is not appropriate for use in routine due to its complexity. The IBD-Disk was then developed, based on the IBD-DI, but has not been validated yet.

The strengths of the VALIDate study will rely on its rigorous, prospective and multicentric design, with a large sample of IBD patients, allowing us to foresee a good statistical power for required analyzes. Apart from the aims of this study, all the socio-demographic and clinical information collected will constitute a valuable database, including data about regimen, sporting activity, medication use (symptomatic and anxiolytic/antidepressant treatments), professional and family status. Moreover, this study will provide interesting comparisons between Physician Global Assessment (PGA) and Patient Global Assessment (PtGA) in a large French cohort of IBD patients (CD and UC), while most of the few studies which analyzed the gaps between physicians’ and patients’ perspectives until now were not conducted specifically in French patients [38–40, 47].

The main practical issue of the study will be patients’ follow-up. Indeed, in order to assess the variability of the IBD Disk over time, patients will be asked to complete again the questionnaires between 3 and 12 months after baseline, during a routine follow-up visit. This will require an efficient “tracking” of included patients, in order to minimize the number of lost to follow-up patients. For that purpose, the person who will manage the electronic dataset (CLB) will also collect the date of the follow-up visit for each patient. However, it seems inevitable that some patients will change the date and others will miss their appointment. To reduce this attrition bias, we chose to increase the sample size at a minimum of 400 patients, while a formula based on feasibility criteria suggested a sample of 100 patients [59]. The same problem will exist for the test-retest reliability: a sub-sample of 70 patients will be asked to complete again the IBD Disk 7 days after baseline visit and to send it by post. In the same way, it is expected that a high proportion of this sub-sample forget to send the questionnaire again. In order to minimize this risk, a phone contact may be made to the patient at the end of the 7-day period.

To conclude, the VALIDate study should be the first study to validate the IBD Disk in a large cohort of French IBD patients. If this instrument proves to be valid, reliable, and reproducible, its use should probably be extended all across France and to other countries, as part of PROs’ assessment in daily practice. Successful dissemination of this tool to other countries will nonetheless require a cultural adaptation and translation process in other languages [60].

## Supplementary information

**Additional file 1.** Annex 1. Letter information for patients.

**Additional file 2.** Annex 2. IBD Disability Index questionnaire (French version).

**Additional file 3.** Annex 3. The IBD Disk questionnaire and the scoring disk (French version).

**Additional file 4.** SPIRIT checklist. SPIRIT_Filled-checklist_VALIDate.

## Data Availability

The datasets used and/or analyzed during the current study are available from the corresponding author on reasonable request.

## Abbreviations

ANOVA: Analysis of Variance
CD: Crohn’s Disease
CRF: Case Report Form
CRP: C-Reactive Protein
FACIT-F: Functional Assessment Chronic Illness Therapy–Fatigue
HAD-A: Hospital And Depression – Anxiety subscale
HAD-D: Hospital And Depression – Depression subscale
HBI: Harvey Bradshaw Index
IBD: Inflammatory Bowel Diseases
IBD-DI: Inflammatory Bowel Diseases Disability Index
IBDQ: Inflammatory Bowel Disease Questionnaire
ICC: Intra-class Correlation Coefficient
ICF: International Classification of Functioning, Disability and Health
IOIBD: International Organization for the Study of Inflammatory Bowel Diseases
PGA: Physician Global Assessment
PtGA: Patient Global Assessment
PROs: Patient-Reported Outcomes
ROC: Receiver Operating Characteristic
SD: Standard Deviation
SIBDQ: Short Inflammatory Bowel Disease Questionnaire
UC: Ulcerative Colitis
VAS: Visual Analog Scale
