# Supplementary material for: Protocol of a multicentric prospective cohort study for the VALIDation of the IBD-disk instrument for assessing disability in inflammatory bowel diseases: the VALIDate study
Source: BMC Gastroenterol. 2020 Apr 16;20:110. doi: 10.1186/s12876-020-01246-7 (PMC7164208; doi:10.1186/s12876-020-01246-7)
Supplement: Supplementary file 1 — Additional file 1. Annex 1. Letter information for patients. [file 12876_2020_1246_MOESM1_ESM.pdf]

Note d'information pour la participation à la recherche

« Etude de validation de l'IBD-Disk pour l'évaluation du handicap fonctionnel des patients atteints de maladies inflammatoires chroniques intestinales »

Titre abrégé : « Validate»

Promoteur : CHU de Nantes
RC18_0032

Investigateur
Nom : Dr Caroline TRANG
Service : Service d'hépato-gastro-entérologie et assistance nutritionnelle
Institut des Maladies de l'Appareil Digestif
Adresse : 1 place Alexis Ricordeau
Téléphone : 02 40 08 31 52

Promoteur de la recherche
Nom : CHU de Nantes
Adresse : 5, allée de l'île Gloriette, 44 093 NANTES
Principaux contacts : Secrétariat du Département Promotion
Téléphone : 02 53 48 28 35

Ce document est remis à la personne participant à la recherche
Un exemplaire est conservé dans le dossier médical

Madame, Monsieur,

Le Centre Hospitalier et Universitaire de Nantes souhaite mener une recherche dont il est le promoteur (c'est à dire qu'il en est responsable et qu'il l'organise). Plus exactement le service de gastroentérologie souhaite valider l'un des questionnaires qui vous sont remis lors de votre consultation de suivi de votre MICI (Maladie Inflammatoires Chronique de l'Intestin) : l'IBD Disk. Pour ce faire nous récupérerons vos réponses à cette consultation et entre 6 mois et 12 mois après. Pour certains (70 patients) nous leur demanderons 3 à 7 jours après de renvoyer ce questionnaire (lettre timbrée fournie) pour vérifier la stabilité de ce questionnaire.Cette recherche est réalisée à partir de données médicales collectées au cours de votre prise en charge et grâce aux réponses que vous apporterez au(x) questionnaire(s) qui vous seront remis.

Cette recherche est réalisée en collaboration avec le CHU d'Angers et le CHU de Rennes.

Votre participation à la recherche, au cas où vous donneriez votre accord, ne pourra vous être confirmée qu'à la condition que vous remplissiez tous les critères d'inclusion pour participer à cette recherche.

Cette recherche ne présente pas de risque pour votre santé. Les résultats qui en seront issus ne permettront pas d'apporter des informations pertinentes pour votre santé en particulier. Ils favoriseront le développement des connaissances dans le domaine des MICI et devront être confirmés, ensuite, par des études cliniques complémentaires, afin de permettre l'essor de nouvelles méthodes de diagnostic, de nouveaux traitements chirurgicaux ou thérapeutiques.

L'investigateur pourra vous informer, sur votre demande, des résultats globaux de cette recherche.

Tournez SVP =>

Pour être menée à bien, cette recherche nécessite la mise en œuvre d'un traitement informatisé de vos données personnelles afin de permettre d'analyser les résultats. Un fichier informatique comportant vos données va donc être constitué. Par mesure de confidentialité et pour respecter votre vie privée, vos données seront systématiquement codées. Seuls les professionnels de santé personnellement en charge de votre suivi auront connaissance de vos données nominatives. Ces données seront susceptibles d'être exploitées dans le cadre de publications ou de communications.

Conformément à la loi, vous disposez d'un droit d'accès, d'opposition et de rectification des données enregistrées sur informatique, à tout moment, par l'intermédiaire de l'investigateur dont les coordonnées se trouvent au début de ce document. Vous disposez également d'un droit d'opposition à la transmission des données couvertes par le secret professionnel susceptibles d'être utilisées et d'être traitées dans le cadre de cette recherche.

Ces données pourront être utilisées lors de recherches ultérieures exclusivement à des fins scientifiques. Vous pouvez retirer votre consentement à cette utilisation ultérieure ou exercer votre faculté d'opposition à tout moment.

Vous pouvez exercer vos droits d'accès et de rectification auprès du Docteur mentionné au début de ce document.
Cette étude est conforme à la loi « Informatique et Libertés » du 6 janvier 1978 modifiée et entre dans la méthodologie de référence MR003.
Toutes les données recueillies au sein des centres associés seront codées dans un fichier Excel avant d'être colligées au sein du CHU de Nantes.

Ce projet ainsi que le présent document ont été présentés au Groupe Nantais d'éthique dans le domaine de la Santé GNEDS.

Vous êtes libre d'accepter ou de refuser de participer à la recherche qui vous est présentée.

- Si vous acceptez, vous êtes libre de changer d'avis à tout moment sans avoir à vous justifier et votre décision ne portera aucun préjudice à la qualité de votre prise en charge. Dans ce cas, les données obtenues avant que votre consentement n'ait été retiré pourront être utilisées, sauf opposition expresse de votre part. Les données recueillies après le retrait de votre consentement ne seront pas utilisées pour cette recherche et resteront destinées à l'usage strict du soin.

- Si vous refusez de participer, les données ne seront pas utilisées pour cette recherche et resteront destinées à l'usage strict du soin.

L'investigateur qui vous a proposé la recherche et vous a donné oralement toutes les informations nécessaires peut répondre à toutes vos questions.

MERCI de conserver cette feuille d'information
