# Supplementary material for: Protocol of a multicentric prospective cohort study for the VALIDation of the IBD-disk instrument for assessing disability in inflammatory bowel diseases: the VALIDate study
Source: BMC Gastroenterol. 2020 Apr 16;20:110. doi: 10.1186/s12876-020-01246-7 (PMC7164208; doi:10.1186/s12876-020-01246-7)
Supplement: Supplementary file 2 — Additional file 2. Annex 2. IBD Disability Index questionnaire (French version). [file 12876_2020_1246_MOESM2_ESM.pdf]

IBD DISABILITY INDEX

Pt Number: ________________________Date:_________________

**VEUILLEZ LIRE CES INSTRUCTIONS À HAUTE VOIX AU PATIENT.**   **La première question porte sur l'état de santé général du patient, elle porte à la fois sur la santé physique et la santé mentale.**

**REPONSES : 0 = Très bon ; 1=Bon ; 2=Moyen ; 3=Mauvais ; 4=Très mauvais**	0	1	2	3	4
**État de santé**					
1. Dans l'ensemble, comment trouvez-vous votre état de santé aujourd'hui ?					

VEUILLEZ LIRE CES INSTRUCTIONS À HAUTE VOIX AU PATIENT   Je vais maintenant passer en revue différentes fonctions de votre corps et différentes activités de votre vie quotidienne. Lorsque vous répondrez à ces questions, vous devrez penser à la semaine passée en tenant compte aussi bien des bons jours que des mauvais jours. Lorsque la question porte sur des difficultés / problèmes que vous avez pu rencontrer en réalisant une activité habituelle, je souhaite que vous teniez compte de l'ampleur de ces difficultés / problèmes en moyenne sur la semaine passée. Par « difficulté », j'entends que vous avez dû faire un effort plus important, que vous avez été gêné(e) ou que vous avez eu mal, ou que vous avez réalisé l'activité plus lentement que d'habitude, ou encore que vous avez modifié votre façon de réaliser cette activité par rapport à l'habitude. Répondez à cette question en tenant compte de l'aide dont vous disposez.   (Lire et montrer l'échelle au patient)

**REPONSES : 0 = Aucun problème; 1=Problèmes légers ; 2=Problèmes modérés ; 3=Problèmes importants ; 4= Problèmes extrêmement importants**	0	1	2	3	4
**Sommeil et énergie**					
2. Globalement, au cours de la semaine passée, avez-vous rencontré des problèmes de sommeil (par ex. problèmes pour s'endormir, réveils nocturnes fréquents ou réveil trop matinal? Et, si oui, de quelle ampleur ? b134					
3. Globalement, au cours de la semaine passée, avez-vous eu des problèmes parce que vous ne vous sentiez pas frais et dispos (fraiche et dispose) pendant la journée (par ex. sensation de fatigue, manque d'énergie) et, si oui, de quelle ampleur ? b130					
**Affect**					
4. Globalement, au cours de la semaine passée, avez-vous rencontré des problèmes parce que vous vous sentiez triste ou déprimé(e) et, si oui, de quelle ampleur ? b152					
5. Globalement, au cours de la semaine passée, le fait de vous sentir inquiet ou anxieux (inquiète ou anxieuse) vous a-t-il posé des problèmes et, si oui, de quelle ampleur? b152					
**Image du corps**					
6. Globalement, au cours de la semaine passée, votre apparence physique ou l'aspect de certaines parties de votre corps vous ont-ils posé des problèmes et, si oui, de quelle ampleur? b1801					
**Douleur**					
7. Globalement, au cours de la semaine passée, avez-vous ressenti des douleurs à l'estomac ou au ventre et, si oui, de quelle ampleur? b28012					

**REPONSES : 0 = Aucune difficulté ; 1= Difficultés légères ; 2=Difficultés modérées; 3=Difficultés importantes; 4=Difficultés extrêmement importantes/ impossibilité**	0	1	2	3	4
**Régulation de la défécation**					
8. Globalement, au cours de la semaine passée, avez-vous rencontré des difficultés pour coordonner et gérer votre défécation, notamment pour choisir un endroit approprié, vous y rendre et vous nettoyer ensuite et, si oui, de quelle ampleur? d5301					
9. Globalement, au cours de la semaine passée, avez-vous rencontré des difficultés pour prendre soin de votre santé au sens large (faire attention à votre santé, votre alimentation, votre activité physique, et votre confort) si oui, de quelle ampleur? d570					
**Activités interpersonnelles**					
10. Globalement, au cours de la semaine passée, avez-vous rencontré des difficultés dans vos relations personnelles et, si oui, de quelle ampleur? d7					
11. Globalement, au cours de la semaine passée, avez-vous rencontré des difficultés pour participer à la vie sociale et, si oui, de quelle ampleur? d920					
**Travail et éducation (veuillez répondre à la question 12a ou 12b en fonction de votre situation)**					
12a. Globalement, la semaine passée, avez-vous eu des difficultés pour travailler et /ou réaliser certaines activités à votre domicile (Tâches ménagères, bricolage, jardinage…) et, si oui, de quelle ampleur ? d840-d859					
12b. Globalement, au cours de la semaine passée, avez-vous rencontrez des difficultés à l'école ou dans vos études et, si oui, de quelle ampleur? d810-d899					

**REPONSES : 0 = 0 ; 1= 1-7; 2 = 8-18; 3 = 19-29; 4 = >29**	0	1	2	3	4
13. Nombre de selles liquides ou très molles au cours de la semaine passée :					

**REPONSES : 0=Non ; 4=Oui ou incertain**	0	N/A	N/A	N/A	4
14. Souffrez-vous d'arthrites ou d'arthralgies ?					

**Total score = S*100/nx4** **n=nombre de questions répondues** **S= somme des scores des n questions** **S est possible si (14-n)/14<20%**	**Total score :……….. (0= pas d'handicap, 100 = niveau le plus élevé d'handicap)**

N/A: non applicable

IBD Disability Index_V2_FR_20141230
