# Supplementary material for: Protocol of a multicentric prospective cohort study for the VALIDation of the IBD-disk instrument for assessing disability in inflammatory bowel diseases: the VALIDate study
Source: BMC Gastroenterol. 2020 Apr 16;20:110. doi: 10.1186/s12876-020-01246-7 (PMC7164208; doi:10.1186/s12876-020-01246-7)
Supplement: Supplementary file 3 — Additional file 3. Annex 3. The IBD Disk questionnaire and the scoring disk (French version). [file 12876_2020_1246_MOESM3_ESM.pdf]

Questionnaire et disque de score

Nom du patient | Numéro | Date

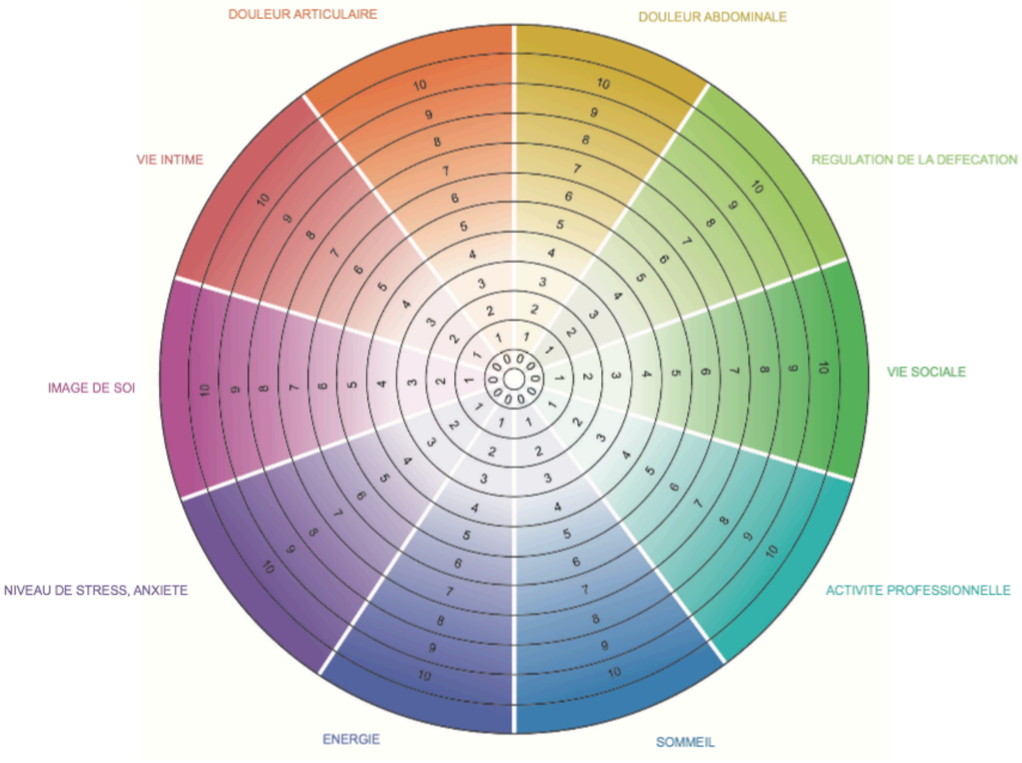

Evaluer (de 1 à 10) et entourer sur le disque votre niveau d'accord pour chacun des items

0	1	2	3	4	5	6	7	8	9	10
Absolument pas d'accord				Ni d'accord, ni pas d'accord					Absolument d'accord	

Score Total: __________

Indiquez ici le score cumulé des 10 items

Au cours des 7 derniers jours :

**DOULEUR ABDOMINALE**	J'ai eu des douleurs au niveau de l'abdomen et/ou de l'estomac
**REGULATION DE LA DEFECATION**	J'ai eu des selles impérieuses que j'ai eu du mal à gérer, trouver des toilettes à temps a été un problème et j'ai parfois eu des difficultés d'essuyage/nettoyage.
**VIE SOCIALE**	J'ai eu des difficultés dans ma relation aux autres et/ou des difficultés d'intégration dans la communauté
**VIE PROFESSIONNELLE**	J'ai eu des difficultés dans mes activités professionnelles, ou dans mes études ou dans la réalisation de tâches quotidiennes liées à la gestion de la maison
**SOMMEIL**	J'ai des difficultés de sommeil, par exemple des problèmes d'endormissement, des réveils nocturnes fréquents ou des réveils très matinaux sans possibilité de rendormissement
**ENERGIE**	Je ne me suis jamais senti(e) véritablement reposé(e), j'ai manqué d'énérgie, je me suis senti(e) fatigué(e)
**NIVEAU DE STRESS, ANXIETE**	Je me suis senti(e) triste, mon moral a été bas, ou je me suis senti(e) déprimé(e) et/ou inquiet et/ou anxieux
**IMAGE DE SOI**	Je n'aime pas mon corps ou certaines parties de mon corps
**VIE INTIME**	J'ai eu des difficultés d'ordre psychologique et/ou physique dans ma sexualité
**DOULEUR ARTICULAIRE**	Mes articulations me font souffrir
